# Cross-Linked Hydrogel for Pharmaceutical Applications: A Review

**DOI:** 10.15171/apb.2017.064

**Published:** 2017-12-31

**Authors:** Rabinarayan parhi

**Affiliations:** GITAM Institute of Pharmacy, GITAM University, Gandhi Nagar Campus, Rushikonda, Visakhapatnam-530045, Andhra Pradesh, India.

**Keywords:** Hydrogel, Cross-linking, Thermoreversible gel, Polymers

## Abstract

Hydrogels are promising biomaterials because of their important qualities such as biocompatibility, biodegradability, hydrophilicity and non-toxicity. These qualities make hydrogels suitable for application in medical and pharmaceutical field. Recently, a tremendous growth of hydrogel application is seen, especially as gel and patch form, in transdermal drug delivery. This review mainly focuses on the types of hydrogels based on cross-linking and; secondly to describe the possible synthesis methods to design hydrogels for different pharmaceutical applications. The synthesis and chemistry of these hydrogels are discussed using specific pharmaceutical examples. The structure and water content in a typical hydrogel have also been discussed.

## Introduction

The formulations applied onto the skin surface are broadly classified in to two groups such as topical and transdermal. Topical formulations deliver drug to local area of skin without systemic exposure. On the other hand, transdermal formulations applied to the skin surface for the purpose of delivering and maintaining effective concentration of drug in the systemic circulation.^1^ There are three basic types of transdermal formulations such as aerosol sprays, semisolids, and patches. Out of these, semisolid transdermal drug formulations such as creams, ointments, and gels are most commonly used to provide systemic effect by delivering the drug across the skin. Gel, among all, is most preferred because of their excellent appearance, fast drug release, desired consistency, ease of manufacturing and quality assessment, and considerable stability.^2^ Most importantly, gels can be modified in order to make it suitable for delivering drug in sustained manner.

An ideal gel should satisfy the following three salient features:^3^

- They must have at least two components i.e. the gelling agent and a fluid component.
- Each component should be continuous throughout the system.
- They should exhibit mechanical properties of the solid state.

Gels are transparent or translucent semisolid formulations containing a high ratio of solvent/ gelling agent. In other word, gel can be defined as a semi-solid preparation composed of low concentrations (<15%) of gelator molecules.^4^ According to USP, gels are defined as a semisolid, either suspension of small inorganic particles or large organic molecules interpenetrated with liquid.^5^ In gel system a liquid phase is constrained within a rigid three dimensional polymeric network thereby exhibiting visco-elastic nature. Gels can be classified into hydrogels and organogels based on the liquid medium entrapped within the polymeric network. Hydrogels are composed of aqueous phase, whereas organogels are composed of aqueous phase along with organic phase.

Hydrogels are hydrophilic, three dimensional cross-linked polymer systems capable of imbibing large amounts of water or biological fluids between their polymeric chains to form aqueous semi-solid/solid gel networks.^6,7^ Polymer networks in hydrogel may absorb water from 10–20% (an arbitrary lower limit) up to thousands of times their dry weight.^8,9^

Followings are the main features of hydrogels for which they are widely used in pharmaceutical applications:^10-14^

- Both compositionally (such as glycosaminoglycans) and mechanically, hydrogels are similar to the native extracellular matrix, thus can serve as a supporting material for cells during tissue regeneration and carrier in delivering a therapeutic agent.
- It exhibits soft material nature, which encourages uptake of water and thereby forming hydrated yet solid materials, just like cells in the body.
- The elastic property (due to the presence of meshes of the networks) of fully swollen or hydrated hydrogels is found to be minimizing irritation to the surrounding tissues after implantation.
- Their hydrophilic and cross-linked property imparts excellent biocompatibility.
- Low interfacial tension between the hydrogel surface and body fluid minimizes protein adsorption and cell adhesion, which reduces the chances of a negative immune reaction.
- Mucoadhesive and bioadhesive characteristics of many polymers used in hydrogel preparations (e.g. polyacrylic acid (PAA), polyethylene glycol (PEG), and polyvinyl alcohol (PVA)) enhance drug residence time on the skin/plasma membrane, leading to increase in tissue permeability.

## Structure and water content in hydrogel

**Figure 1:**
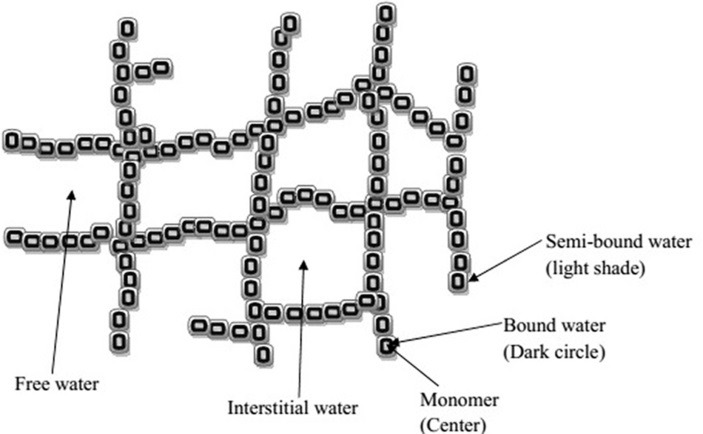
Schematic diagram of molecular structure of hydrogel network with different types of water.

Presence of water in hydrogels plays an important role in the overall permeation of active ingredients into and out of the gel. Water can be associated to any hydrogel structure in following ways as shown in Figure 1.

The types of water associated with hydrogel are:

- Bound water (primary and secondary)
- Semibound water
- Interstitial water
- Free water or bulk water

When a dry hydrogel comes in contact with water, absorption of water into its matrix starts. At first water molecules entering the matrix will directly attach to the hydrophilic groups, forming hydrophilic bound water or primary bound water. The polymeric network swells because of complete hydration of the polar groups that resulted in the exposer of hydrophobic groups. These exposed groups also interact with water molecules, leading to hydrophobically-bound water, or ‘secondary bound water’. The combination of primary and secondary bound water is often called the bound water. This water is considered as integral part of hydrogel structure and can only be separated out from the hydrogel under extreme conditions. After the saturation of hydrophilic and hydrophobic groups, the additional water that is absorbed due to the osmotic driving force of the network chains is called free water or bulk water. Between the bound water, present on polymeric monomer surface, and free water, there is a presence of water layer called semibound water. Interstitial water is present in the interstices of hydrated polymeric network that is trapped physically but not attached to hydrogel network.^7,15^

## Classification of hydrogel

**Figure 2:**
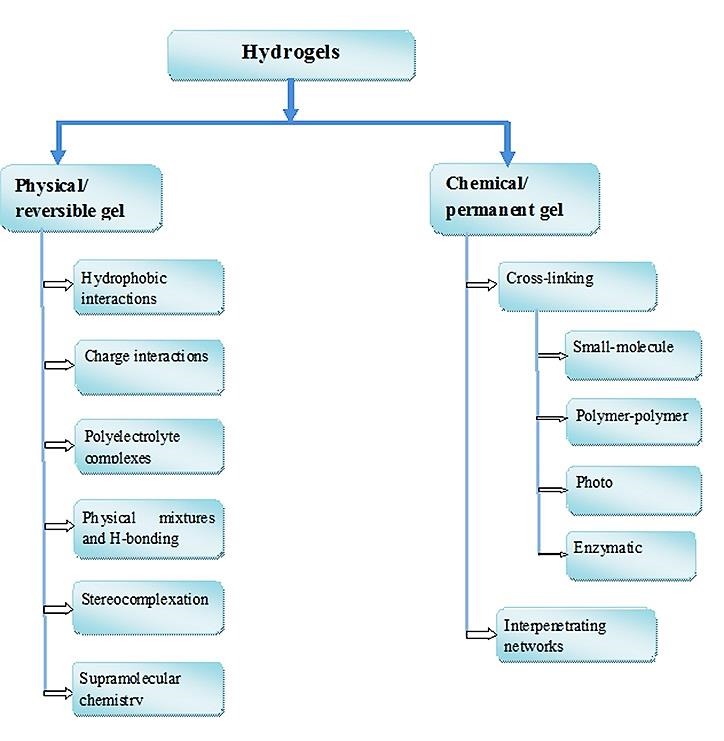
Classification of hydrogels based on cross-linking

Pharmaceutical hydrogels may be classified on the basis of their type of cross-linking or microstructure into following types (Figure 2):

### Physical or reversible gel

In the preparation of physical gel, the selection of polymer is crucial and it depends on the two primary criteria; interaction among the chain must be strong enough to form semi-permanent junction in the molecular network and the network should hold large amount of water molecules inside it. The forces involved in the physical gel formation are hydrophobic, electrostatic, and hydrogen bonding between polymer chains.^16,17^ Because the network formation by all of these interactions is purely physical, gel formation can be reversed.

#### Hydrophobic interactions

The mechanism involved in the sol-gel and gel-sol conversion of thermoreversible gel is based on hydrophobic interaction. The polymers showing hydrophobic interaction must be having both hydrophilic and hydrophobic domain, called as amphiphiles. Such amphiphiles are water soluble at low temperature. However, following increased temperature the hydrophobic domains (gelators) aggregate to minimize the hydrophobic surface area contacting the bulk water. This reduces the amount of structured water surrounding the hydrophobic domains and maximizing the solvent entropy. The gelation temperature depends on various parameters such as the concentration of the polymer, the length of the hydrophobic block, and the chemical structure of the polymer.^18^

**Figure 3:**
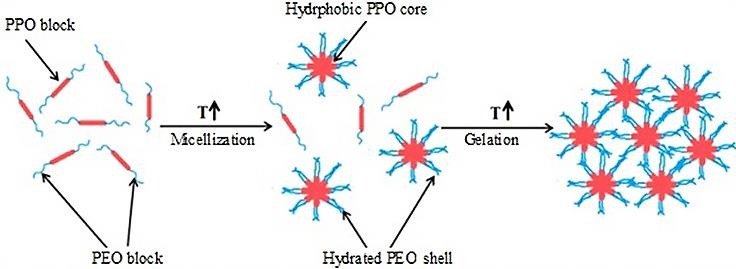
Schematic representation of physical gelation mechanism of PX 407 in water (Modified from reference 20)

Poloxamer (PX) is such a polymer which shows thermoreversible property in aqueous solutions. PX consists of hydrophilic poly (ethylene oxide) (PEO) and hydrophobic poly(propylene oxide) (PPO) blocks arranged in a tri-block structure as PEO–PPO–PEO [19]. The gelation of PX depends upon both temperature and concentration and the total gelling process is typically divided into two steps (Figure 3). The first step occurs when the temperature is increased to reach the critical micelle temperature (CMT) and the PX co-polymers aggregate to form spherical micelles. These micelles consist of an outer shell of hydrated swollen PEO chains with dehydrated PPO blocks as the core. The process develops into the second step when a further increase in the temperature packs the micelles in an orderly manner to form gels.^19^ Gelation is also dependent on the concentration of PX molecules in solution. At very low concentrations, PX molecules exist as monomers in solution. Increasing PX concentration up to 10^−4^% to 10^−5^% (w/w) leads to a critical micelle concentration (CMC), where spherical micelles started to built up. Further increase in PX concentration leads to a tightly packed system with a gel consistency.^20,21^ Between 20-30% (w/v) aqueous solution of PX 407 at cold temperature (4-5°C) forms clear liquid and get transformed to gel at body temperature (37°C).

Release of the model drug benzoic acid was found to be decreased with the increase in PX 407 concentration. This is probably due to an increase in size and number of micelles leading to decrease in size and number of aqueous channels. Further the modification of benzoic acid to more lipophilic moiety p-hydroxybenzoic acid causes a decrease in its release rate. This could be due to the greater partitioning of lipophilic derivatives into the micellar region within the gel structure.^22^ Erukova* et al.* (2000) observed that PX facilitated the permeation of comparatively large molecules, including 2-n-undecylmalonic acid and doxorubicin, across lipid bilayers, whereas the permeation of small solutes (such as ammonium and acetic acid) remained unaffected. They also observed that PX accelerated the translocation of large hydrophobic anions (tetraphenylborate). They attributed the above effect of PX with the content of PEO units i.e. it is enhanced when the portion of PEO block in the copolymer is increased.^23^ Gels and niosomal gels of meloxicam were developed using polymers such as PX 407, Chitosan and Carbopol-934. The PX 407 gel or niosomal PX 407 gel showed the superior drug release over the other formulations. Between the two types of gel of PX 407, niosomal gels exhibited superiority over conventional gels for anti-inflammatory test.^24^ Thermosensitive gels of drugs such as indomethacin,^25^ adriamycin and 5-flurouracil,^26^ mitomycin C,^27^ and ketoprofen^28^ were successfully developed and studied. The gel formed from PX alone (as in case of above) leads to relatively rapid diffusion of drugs out of the hydrophilic gel matrix by the dilution of water that entered the gel matrix.^29^ To circumvent above drawback many researchers have used combination of polymers to reduce the fast entry of water and subsequently diffusion of drug out of gel structure. In addition, it will retain the gel structure for longer period of time and thereby sustain the drug release.

Based on the above fact thermoreversible gels of meloxicam were prepared using PX 407 and PX 188 (20-30% w/w) in combination with different additives such polyvinylmethylether maleic anhydride copolymer, HPMC, PE-400, DMSO, sodium chloride. Among investigated gel bases, PX 407-HPMC gel was found to be ideal due to its gel strength (1.560±0.0135 N), viscosity (312.3±2.06 cP) and release characteristics.^30^ HPMC K100M (2% w/v) and PX 407 (20% w/v) based procaine bioadhesive gels were prepared and the drug release was found to be increased with the increase in drug concentration from 1.5% to 3.5% (w/w) in the gels and the temperature of surrounding solutions from 28°C to 42°C.^31^ Pranoprofen release from HPMC and PX 407 based bioadhesive gel was increased with the increase in temperature, which was attributed to increased in activation energy of the drug.^32,33^ Similarly, Shin *et al.* (2004) successfully prepared lidocaine HCl gel using HPMC (2% w/v) and PX 407 (20% w/v) for analgesic activity.^34^ We have successfully prepared and characterized bioadhesive hydrogels of aceclofenac and metoprolol succinate containing various concentrations of HPMC (0.5-4% w/w) and PX 407 (15-30% w/w).^35,36^

A range of other synthetic amphiphilic thermally gelling polymers have been investigated. Based on the position of hydrophilic and hydrophobic blocks they may be classified in to two categories; symmetric and asymmetric. Symmetric means the presence of hydrophobic block at the center and hydrophilic blocks on either side and vice versa. Examples of this category are PX (discussed in the previous section), PEG- poly (lactide-co-glycolide) (PLGA)-PEG^37^ or PLGA-PEG-PLGA^38^ etc. Compared to PX systems, PL(G)A-based triblock gelators exhibit better biodegradability, higher gelation temperatures (which is allowing easier handling of injectable preparation), and sustained drug release.^39^ Another example of symmetric triblock polymer is Poly (N-isopropylacrylamide) (PNIPAM)-poly (phosphorylcholine)-PNIPAM, which form gels at 6-7 wt % when the temperature exceeded 32°C (is the phase transition temperature of PNIPAM).^40^ PNIPAM grafted with other polymers such as hyaluronic acid and chitosan were developed successfully for the sustained release of riboflavin^41^ and 5-fluorouracil,^42^ respectively. Asymmetric triblock copolymer formed of PEG, PLA, and poly (L-glutamic acid) were also developed and observed that the L-glutamic acid block permits selective modification with specific targeting groups such as the cell-adhesive RGD peptide.^43^

Reverse thermal gelation is also evident from some natural polymers. A biocompatible, parenteral gel composed of chitosan solutions and glycerol-2-phosphate was prepared and the gel was found to be exhibited sol-gel transition at a temperature close to 37°C.^44^ Chitosan grafted with PEG (approximately 40 wt %) based thermoreversible gel was prepared and studied for bovine serum albumin (BSA) release. This gel showed an initial burst release for 5 h and afterwards a steady linear release of BSA for a period of approximately 70 h.^45^ Liang *et al.* (2004) successfully used thermosensitive methylcellulose to thermally gel aqueous alginate solution blended with various salts such as CaCl_2_, Na_2_HPO_4_, and NaCl for the delivery of BSA.^46^ Similar thermally triggered transitions have also been exhibited by hydroxypropylcellulose.^47^

#### Charge interactions

**Figure 4:**
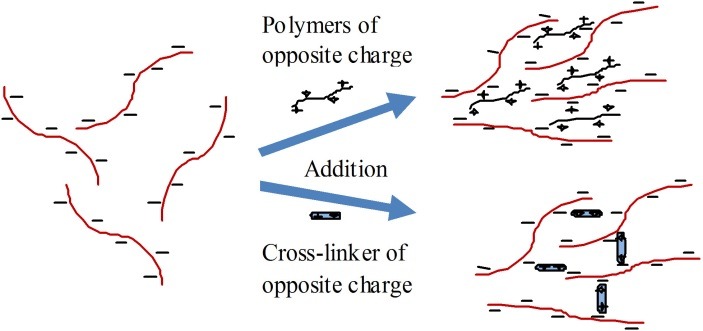
Schematic illustration of mechanism of charge interactions with an oppositely charged polymer and a cross-linker

Charge interactions which lead to the formation of hydrogel may occur in two ways; (i) between a polymer and an oppositely charged small molecule as linker, and (ii) between two polymers of opposite charge (Figure 4). This type of cross-linking (or decross-linking) can be triggered by pH changes that ionize or protonate the ionic functional groups. These hydrogels can be tailored to achieve a specific set of thermal, mechanical and degradation profiles. In some cases they can formed in situ, known as in situ gelling hydrogels.^48^ A mixture of quaternized chitosan (N-[(2-hydroxy-3-trimethylammonium) propyl] chitosan chloride (HTCC)) and glycerophosphate (GP) forms an ionically cross-linked gel at 37°C which can release doxorubicin hydrochloride as a function of pH.^49^ At acidic condition and physiological condition these hydrogel dissolved and released drug quickly, while it released drug slowly at neutral or basic conditions.

The charged interactions have been widely investigated for cross-linking in situ gelling hydrogels. The main advantage of this interaction is that biodegradation can occur as ionic species in extracellular fluid which bind competitively with the gel components and as a result decross-linking of network happen. In addition, this approach can also be used to cross-link microparticle or nanoparticle gels to equip it with suitable drug delivery properties. For instance anionic (methacrylic acid (MAA)) and cationic (dimethylaminoethyl methacrylate (DMAEMA)) polymers coated hydroxyethyl methacrylate-derivatized dextran microspheres exhibit spontaneous gelation upon mixing due to ionic complex formation between the oppositely charged microparticles.^50^ Another approach, so called ionic-complementarity, uses peptide of alternating positive and negative charge distribution leading to peptide self-assembly. These peptides assume β-sheet secondary structure predominantly, and can form hydrogels. The advantage of this approach is that nanoscopic and/or macroscopic structures with great stability and functionality can be developed by varying peptide concentration, pH, presence of salts, and time.^51^

#### Polyelectrolyte complexes (PECs)/ Complex coacervate gel 

**Figure 5:**
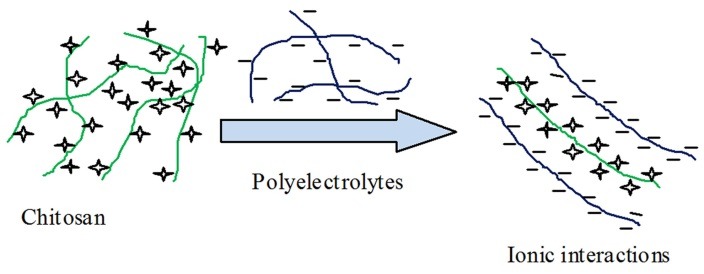
Schematic diagram of PEC mechanism

PECs are formed by the electrostatic interactions between two oppositely charged polyelectrolytes in an aqueous solution as shown as in Figure 5. Thus both the polymers must be ionized and have opposite charges. This implies that the reaction can only occur at pH values in the vicinity of the pKa interval of the two polymers.^16^ Its distinction from electrostatic interactions is based on the MW of molecules. The PEC is formed between larger molecules with a broad MW range and the association is stronger than other secondary binding interactions like hydrogen bonding (H-bonding) or van der Waals interactions. For example formation of PEC between chitosan, a polycation, with polyanions such as proteins (e.g. collagen, gelatin, albumin), polysaccharides (e.g. alginate, pectin or xanthan) and synthetic polymers (e.g. PAA) were reported.^52^

Chitosan based PEC of metoprolol tartrate were developed in the form of beads and hydrogels by interaction of cationic chitosan with anionic sodium alginate, carboxymethyl cellulose sodium and k-carrageenan using simple ionic gelation technique. Among all the combinations, PEC of chitosan and carboxymethylcellulose sodium exhibited least in vitro drug release and was able to extend the release of metoprolol tartrate up to 12 h.^53^ Another chitosan based PEC hydrogel with improved viscoe-lastic behavior was developed using low concentration (0.1 to 0.8%) of chitosan and constant gelatin concentration of 1%.^54^ Chang *et al.* (2014) successfully developed PEC of chitosan and poly-(γ-glutamic acid) (γ-PGA) to promote new bone formation in the alveolar socket following tooth extraction.^55^ With same composition, a cytocompatible PEC was developed for biological application.^56^ A PEC composed of alginate and chitosan was developed and found to be having improved thermal, chemical, and mechanical properties and stability.^57^ Sometimes, same polymers having opposite charges, fraction is modified to opposite charge by chemical processes, can also be used to prepare PEC hydrogel. One of such hydrogel was developed with phosphorylated chitosan (a polycation) and chitosan (a polyanion) as an osteoblast carrier. These hydrogels exhibited three-dimensional hierarchically-porous structure and good cyto-biocompatibility for osteoblasts in vitro.^58^ Based on above approach, another novel PEC encapsulated dexamethasone hydrogel was prepared using polycationic N-trimethyl chitosan and polyanionic N-carboxymethyl chitosan to target distal intestine.^59^ Chitosan, in many drug delivery systems, present as unionized amine form at the neutral/alkaline pH of physiological application sites, while the anionic polyelectrolyte is in the ionized form. In this form to stabilize any PEC is a challenging task, but it can be overcome by using modified chitosan such as quaternized chitosan. This modified chitosan along with PAA were used to prepare PEC hydrogels with improved mechanical properties by in situ polymerization of acrylic acid monomers in the concentrated quaternized chitosan solution.^60^

The advantage of PEC is that it is formed without the use of organic precursors, catalysts, or reactive agents, avoiding the concern about safety in the body. During complexation, polyelectrolytes can either coacervate, or form a more or less compact hydrogel. However, if ionic interactions are too strong, precipitation can occur,^61^ which is quite common and hinders the formation of hydrogels. Precipitation can be prevented weakening the electrostatic attraction by the addition of salts, such as NaCl.^16^

#### Physical mixtures and hydrogen bonding interaction

**Figure 6:**
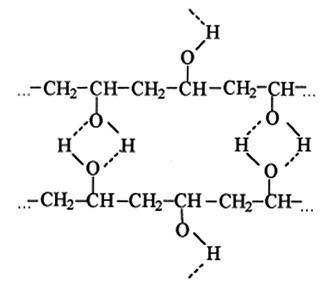
Hydrogen bonding in PVA solutions

Hydrogels by H-bonding interactions can be formed by freeze-thaw cycles using PVA alone^62^ or in combination with other polymers such as chitosan.^52^ In the earlier case crystallization plays a big part in the creation of cross-links between PVA chains during lyophilization. The cross-linking is possible due to the existence of regular pendant hydroxyl groups on PVA that are able to form crystallites by strong inter-chain H-bonding. The crystals formed by this method are generally considered to have a two-molecule monoclinic unit cell, with hydroxyl groups randomly placed on either side of both polymer chains as shown in Figure 6. It has been proposed that in the first freeze-thaw cycle, the initial freezing of water, and associated expansion, leads to increased polymer concentration in the unfrozen phase. Crystallization can then take place within the polymer-rich microphages.^63^

Processing parameters such as the concentration, the number of thermal cycles, and the thawing rate affect the mechanical behavior of cryogel. Pazos *et al.* (2009) reported that with increasing number of thermal cycles resulting in the formation of the strongest cryogels because the number of intermolecular hydrogen bonding increases and molecular chains aggregate.^64^ They also reported that the increase in thawing rate from 2 (0.333°C/min) to 5 (0.067°C/min) exhibited the same effect on the gel’s elastic properties. Meenakshi, and Ahuja, (2015) found that 4 freeze-thaw cycles are optimum for the preparation of composite cryogels of metronidazole with carboxymethyl tamarind kernel polysaccharide and PVA. This cryogel provided 75.77% of drug release over a period of 6h and had better thermal stability.^65^

In case of composite cryogel, mixture of polymers forms junction points in the form of crystallites and inter-polymer interaction after a series of freeze–thaw cycles.^16^ These interactions act as cross-linking sites of the hydrogel. Addition of another polymer to PVA negatively affects the formation of PVA crystallites thereby forming hydrogels of less ordered structures. Chitosan-PVA polymer blend is one such example of polymer mixture, where increasing chitosan concentration in the blend reduces the crystallinity of hydrogel.^52^ The addition of chitosan decreased maximum strength and thermal stability of PVA cryogel containing minocycline and gentamycin, while it increased the swelling ability, elasticity and porosity of cryogel.^66,67^ Contrary to above, Paduraru *et al.* (2012) reported that strength of the cryogel increased by increasing the microcrystalline cellulose content from 0 to 50 % wt.^68^

H-bonding can also be formed with blend of two or more natural polymers such as hyaluronic acid-methylcellulose, gelatin-agar, and starch-carboxymethyl cellulose. The formation of H-bonding interactions between the polymer chains along with the compatible geometry resulted in the enhanced visco-elastic properties of the hydrogel.^69^ The main advantage of above mixture of polymers is the excellent biocompatibility offered by them due to the absence of chemical cross-linkers. However, the problem associated with it is the breaking of H-bonded networks over a few hours in vivo due to influx of water, which restricting their use to short-acting drug release systems.^18^

#### Stereocomplexation 

Stereocomplexation is a synergistic interaction between polymer chains or small molecules of the same chemical composition, occasionally with different chemical composition, but different stereochemistry and stereocomplex formation occur due to the interaction between polymers having different tacticities or configurations.^70^ It may be homo-stereocomplexation or hetero-stereocomplexation. In former case the complex formation occurs between same molecules but with different stereochemistry. For example, interaction between polylactide blocks with L- and D-stereochemistry.^70^ Another well known example of stereocomplexation is the one between isotactic and syndiotactic poly(methyl methacrylate) (PMMA).^71^ Hetero-stereocomplexation occurs between two different molecules with different stereochemistry such as D-PLA and L-configured peptides leuproide, a luteinizing hormone-releasing hormone.^72^ Natural polymers such as dextran precursors can be grafted to L-lactide and D-lactide oligomers leading to the spontaneous gelation in water. It provided an excellent biocompatibility and biodegradability.^73^

Like H-bond interactions, stereocomplexation do not need harsh organic solvents and chemical cross-linkers. Main limitation of stereocomplexation is the restricted range of polymer compositions available which can be used i.e. even small changes in the stereochemistry or else changing the composition of matter can significantly weaken or altogether eliminate the stereochemical interaction.

#### Supramolecular chemistry 

Supramolecular chemistry is based on the association of two or more chemical species held together by intermolecular forces. The intermolecular forces includes electrostatic interactions, H-bonding, metal coordination, π- π interaction, Van der Waals forces etc. and the important methods by which supermolecules formed are; molecular self-assembly, folding, host-guest interaction (e.g. inclusion complex), recognization and complexation and mechanical-interlocking.^74^

This approach has wide application such as material technology, catalysis, data storage and processing and in the preparation of medicines. The most common type of cross-linking interaction used to prepare hydrogels is the formation of inclusion complexes. For example, formation of a reversible hydrogel complex between PEO polymers and α-cyclodextrins.^75^ Similarly, β-cyclodextrin can be used to gel PPO-grafted dextran into a hydrogel.^76^ Self-assembled systems have also been reported that apply both hydrophobic interactions and supermolecular chemistry to facilitate the formation of denser and/or more stable hydrogel networks. A self-assembled system, PEO-poly(R-3-hydroxybutyrate) (PHB)-PEO triblock copolymers, was complexed with α-cyclodextrin to form strong hydrogel networks. The cross-linking was accomplished by (a) the thermal gelation of the hydrophobic PHB segments and (b) the inclusion complexes formed between the PEO segments and cyclodextrin. This system is capable of releasing fluorescein isothiocyanate-dextran for up to one month.^77^

#### Chemical or permanent gel 

Physically cross-linked hydrogels are prepared without the use of cross-linking entities or chemical modification. Despite of the above advantage, it is inflexible towards variables such as gelation time, gel pore size, chemical functionalization, and degradation or dissolution, leading to inconsistent performance in vivo.^52^ In contrast, chemically cross-linked hydrogels allows absorption of water and/or bioactive compounds without dissolution and permits drug release by diffusion.^18^

#### Cross-linking 

Chemical cross-linking method uses covalent bonding between polymer chains in order to produce permanent hydrogel. The cross-link formation was carried out by the addition of small cross-linkers molecules, polymer-polymer conjugation, photosensitive agents or by enzyme catalyzed reaction.

#### Small-molecule cross-linking

Preparation of small-molecule cross linking hydrogels at the minimum requires one polymer and a small molecule as cross-linker in an appropriate solvent. Cross-linkers are molecules with at least two reactive functional groups that allow the formation of bridges between polymeric chains.^78^ Small cross-linkers can be of two types; bi-functional molecules or drug molecules. In the former case drug molecule is entrapped within the hydrogel formed due to the interaction between bi-functional molecule and polymer. Moreover, drug molecule having two functional group covalently bonded with polymeric chains to form hydrogel, which avoids the use of cross-linkers. This approach is limited to drug in which two reactive functional groups are available. One such drug molecule is primaquine (a di-amino drug), which has been used to cross-link periodate-oxidized gum arabic into a hydrogel through the rapid formation of Schiff bases between the amine groups and the aldehyde groups present in the drug and polymer, respectively.^79^

The simplest form of the cross-linking takes place between aldehyde and amino groups to form Schiff base. For example, dialdehyde such as glyoxal and particularly glutaraldehyde^46^ forms covalent imine bonds with the amino groups of chitosan via Schiff reaction.^80^ In other cases, dextran-tyramine^81^ and hyaluronic acid-tyramine^82^ covalently bonded by using horseradish peroxidase (HRP) and hydrogen peroxide (H_2_O_2_) as cross-linkers to form hydrogels with improved controllable gelation times ranging from 5 s to 9 min according to the reactant concentrations used. A general disadvantage of each of these small-molecule cross-linking methods is the potential toxicity of residual unreacted cross-linker agents in vivo. For instance, glutaraldehyde and glyoxal are known to be neurotoxic and mutagenic, respectively.^80^

Genipin (a naturally derived chemical from the fruit ofgardenia) widely used as a cross-linking agent alternative to dialdehydes due to its biocompatibility.^83^ It was also reported that genipin bind polymers, such as chitosan and gelatin with biological tissues covalently.^84^ Furthermore, amino-terminated groups containing molecules such as PEG, N,O-carboxymethyl chitosan,^85^ and BSA^86^ have been cross-linked to genipin to form hydrogels with flexible dissolution rates from 3 min to more than 100 days. Even though genipin shows good biocompatibility, it is still liable to negatively interact with encapsulated drugs, an unavoidable problem for gelation in the presence of a therapeutic.

This approach used to develop transdermal drug delivery systems for the delivery of oxprenolol HCl,^87^ propanolol HCl,^88^ 5-fluorouracil,^89^ indomethacin from gel beads for the control release.^90^

#### Polymer-polymer cross-linking or hybrid polymer networks (HPN)

In this type of hydrogel, the cross-linking reaction occurs between a structural unit of a polymeric chain and a structural unit of another polymeric chain of another type. Therefore, polymers must be pre-functionalized with reactive functional groups. Several types of covalent linkages can be made depending on the desired speed of cross-linking, selection of targeted reactive functional groups, and biodegradability of the resulting conjugates.

Widely investigated in situ cross-linking phenomenon is Michael addition between a nucleophile (i.e. an amine or a thiol) and a vinyl group. For example, cross-linking of vinyl sulfone-functionalized dextrans with thiolated PEG. This type of approach provides the flexibility in forming multiple types of bonds, relative biological inertness and finally rapid formation of hydrogel.^91^ Another example is the formation of a hydrazone bond between an aldehyde and a hydrazide which facilitates rapid cross-linking of gel precursors.^92^ Control release of tissue plasminogen activator and budesonide^93^ to the peritoneum was achieved by using hyaluronic acid cross-linking by hydrazone bonds. Similar approaches also been used to design poly (aldehyde guluronate) for the effective delivery of osteoblasts and growth factors.^94^

The major advantages of this approach are the elimination of cross-linker molecules and in situ formation of covalently bonded hydrogels. The main limitation of this approach is the requirement for significant polymeric modification to link functional group to polymeric chain. In addition, the pre-gel polymers are often themselves somewhat cytotoxic, even when prepared from highly biocompatible polymer precursors.

#### Photo cross-linking 

Formation of hydrogels based on photo cross-linking depends on the presence of photo sensitive functional groups. By linking a photo sensitive functional group to a polymer enables it to form cross-linkages upon irradiation with light such as UV light. Chitosan is one such polymer which was studied more compared to other polymers. A photo cross-linked chitosan hydrogel was developed by incorporating azide groups (-N_3_) to polymeric chain of chitosan. After its exposure to UV light, the azide group is converted to nitrene group (R-N:) which binds free amino groups of chitosan leading to the in situ formation of hydrogel within a minute.^95^ Photo cross-linked hydrogel can also be formed between the polymers. One such example is thermo-sensitive chitosan-pluronic hydrogel, where both the polymers were functionalized with photo sensitive acrylate groups (CH_2_=CHCOO−) by UV irradiation. The resulted cross-linked polymer formed a physical network at temperatures above the lower critical solution temperature (LCST) and the same exhibited the ability to release encapsulated human growth hormone (hGH) in sustained manner.^96^ The above combination was also used to deliver plasmid DNA.^97^ Another chitosan-PEG-based hydrogel was developed by modifying chitosan with photoreactive azidobenzoic acid and PEG with arginylglycylaspartic acid peptide. Upon UV illumination, a free-radical photo-initiated polymerization took place that led to the formation of hydrogel in situ. Resulted hydrogel helped in targeting the injured myocardium for the delivery of growth factors and cells.^98^

The advantage of this approach is to facilitate easy and speedy formation of hydrogel. It also offers safety and low cost preparation compared to chemical methods, which generally require the addition of different reactive species, initiators, or catalysts. However, this technique requires a photosensitizer and prolonged irradiation, which could also result in local rise of temperature, thereby damaging neighboring cells and tissue.^99^

#### Enzymatic cross-linking 

This is a new approach to form in situ hydrogel using enzyme-catalyzed cross-linking reaction between polymer chains. A number of enzymes including transglutaminases (TG),^100-102^ peroxidases,^103,104^tyrosinase,^105^ phosphopantetheinyl transferase, lysyl oxidase, plasma amine oxidase, and phosphatases^106^ were studied and reported.

TG belong to thiol group of enzymes which catalyze the formation of highly resistant covalent bonds between a free amine group of a protein or peptide-bound lysine and the g-carboxamide group of a protein or peptide-bound glutamine.^106^ Yung* et al.* (2007) have successfully developed thermally stable and biocompatible gelatin hydrogels cross-linked by microbial TG (mTG), which is capable of delivering encapsulated regenerative cells (HEK293) in a controlled manner.^100^ The same group tested the diffusion of anticancer drug, interleukin-2, from mTG cross-linked gelatin and found that the resulted hydrogels are not only cyto-compatible but also have potential to be used as sustained drug release devices.^101^ Between animal derived tissue TG (tTG) and recombinant human TG (hTG) enzymes used for cross-linking of two classes of protein polymers containing either lysine or glutamine under physiological conditions, tTG completed the cross-linking faster (within 2 min) compared to hTG.^102^

HRP and soy bean peroxidase are the most commonly used peroxidase enzymes in the formation of hydrogel. They catalyze the conjugation of phenol and aniline derivatives in the presence of substrate H_2_O_2_. In this reaction the HRP promptly combines with H_2_O_2_ and the resulted complex can oxidize hydroxyphenyl groups present in compound such as tyramine and tyrosine.^106^ Recently, Kim* et al.* (2011) developed HRP catalyzed injectable tyramine modified hyaluronic acid (HA-Tyr) hydrogels in two steps. In the first step, HA-Tyr conjugate was synthesized by amide bond formation between carboxyl groups of HA and amine groups of tyramine and in the subsequent step HA-Tyr hydrogels were prepared by radical cross-linking reaction using HRP and H_2_O_2_. This hydrogel was used to deliver dexamethasone intra-articularly for the treatment of rheumatoid arthritis.^103^ An enzymatically cross-linked injectable hydrogel was developed from chitosan derivatives, chitosan-glycolic acid, and phloretic acid using HRP and H_2_O_2_.^104^ This hydrogel can be formed (gelation time) from 10 sec to 4 min by using the polymer concentration from 3 to 1% and has potential for cartilage tissue engineering.

Tyrosinases are oxidative enzymes that convert accessible tyrosine residues of proteins (e.g. gelatin) into reactive o-quinone moiety, which can undergo non-enzymatic reactions with available nucleophiles such amino groups of chitosan. Chen *et al.* (2002) developed gelatin gel by two methods namely; (i) cooling the gelatin solution and (ii) tyrosinase-catalyzed hydrogel formation and observed that the second method demonstrated better mechanical properties which can be exploited for medical and industrial applications.^107^ Gelatin-chitosan gels were developed using both TG and tyrosinase for comparison and following observations were made; (i) chitosan was not required for transglutaminase-catalyzed gel formation, although gel formation was faster and the resulted gel was stronger in its presence, (ii) TG-catalyzed gelatin-chitosan gels lost the ability to undergo thermally reversible transitions, (iii) tyrosinase-catalyzed gelatin-chitosan gels were considerably weaker than transglutaminase-catalyzed gels, but can be strengthened by cooling below gel-point of gelatin.^105^

#### Interpenetrating networks (IPNs)

An IPN may be defined as any material that contains two or more polymers in the network form in which one polymer is cross-linked in the presence of other.^108,109^ IPNs are considered as “alloys” of cross-linked polymers, which are formed without any covalent bonds between them. These networks cannot be separated unless chemical bonds are broken.^110^

The polymers to be used in the preparation of IPN hydrogel must fulfill following three conditions; at least one polymer must be synthesized and/or cross-linked within the immediate presence of the other, both polymers should have similar kinetics, and there should not be dramatic phase separation between/among the polymers.^111^ An IPN is different from other polymer combination because it has no viscoelastic property, and it swells, but does not dissolve in any solvent.^112^

Based on the chemistry of preparation, IPN hydrogels can be classified into following types:^113,114^

- Simultaneous IPN: when both the networks are synthesized simultaneously from the precursors at the same time by independent, non-interfering routs.
- Sequential IPN: it is formed by swelling of a single-network hydrogel into a solution containing the mixture of monomer, initiator and activator, with or without a cross-linker.

Sequential IPNs are of two types namely, fully-IPN and semi-IPN based on the presence and absence of cross-linker, respectively.

According to the structure, IPN hydrogels can be classified into three categories:^114^

- IPNs: formed by two juxtaposed networks, with many entanglements and physical interactions between them.
- Homo-IPNs: these are developed from the two polymers which are having independent networks, but have same structure.
- Semi- or Pseudo-IPNs: in this type one polymer has a linear instead of a network structure.

There are mainly two classes of polymers which are used to synthesize IPN hydrogels, including natural polymers and their derivatives such as polysaccharides and proteins, and synthetic polymers containing hydrophilic functional groups (e.g. -COOH, -OH, -CONH_2_, SO_3_H, amines etc).^8^

IPN hydrogels of clarithromycin were synthesized by using three polymers such as chitosan, PAA and poly (vinyl pyrrolidone) (PVP) and two cross-linking agents (e.g. glutaraldehyde and N,N’-methylene-bis-acrylamide). The resulted IPN hydrogels have good mucoadhesion property, which helped the gel to retain in gastric environment of stomach for prolonged period of time. As a result of that IPN hydrogel maintain antibiotic concentration in stomach for prolonged period of time, thereby used as a drug delivery system for treatment of *H. pylori* infection and in management of peptic ulcer.^115^ Semi-IPN hydrogels were synthesized by cross-linking chitosan and PVP blend with glutaraldehyde and found that the resulted semi-IPN gels have potential to deliver clarithromycin in the gastric medium.^116^ Kulkarni *et al.* (2010) developed IPN hydrogel membranes of prazosin hydrochloride using sodium alginate (SA) and PVA as polymers for transdermal delivery. The stiffness of the film was increased by the addition of cross-linker glutaraldehyde and both the stiffness and in vitro drug release property film depend on the concentrations of glutaraldehyde in membranes. The prepared IPN membranes extended prazosin hydrochloride release up to 24h, whereas membranes made up off SA and PVA alone showed fast drug release.^117^ Microspheres of theophylline were successfully prepared based on IPNs method using chitosan and methylcellulose as polymers and glutaraldehyde as a cross-linker. The encapsulation efficiency was found to be up to 82% and the extended drug release up to 12 h.^118^ Likewise, duration of 5-fluorouracil release was significantly sustained for up to 52 h from new thermo-sensitive gels in which a chitosan network is crosslinked with various concentrations of glutaraldehyde that interpenetrates PX gels.^119^

Multicomponent networks as IPNs have better mechanical strength and swelling/deswelling response compared to single-network hydrogels.^120^ Furthermore, the cross-linking density, hydrogel porosity, and gel stiffness can be adjusted in IPN-based hydrogels according to the target application. The drawbacks are: (i) it has a problem with encapsulating a wide variety of therapeutic agents, especially sensitive bimolecular, and IPN, particularly sequential type, (ii) preparation requires the use of toxic agents such as initiator, activator and cross-linker in order to initiate or catalyze the polymerization or to catalyze the cross-linking.^121^ Difference between physical and chemical hydrogel and types of cross-linked hydrogel along with their polymer system and drug incorporated is presented in Table 1 and Table 2, respectively.

**Table 1:** Difference between physical and chemical gel.^8^

**Physical hydrogel**	**Chemical hydrogel**
Physical hydrogels are formed by molecular entanglements, and/or secondary forces including ionic, H-bonding or hydrophobic forces.	Chemical hydrogels formed by covalent cross-linking.
Above bonds are weak, thus physical hydrogels are considered as reversible gel.	These are termed as permanent or irreversible as covalent bonds are strong.
These are prepared without the use of cross-linking entities or chemical modification.	These are prepared using cross-linking entities or chemical modification.
It is inflexible towards variables such as gelation time, gel pore size, chemical functionalization, and degradation or dissolution, leading to inconsistent performance in vivo.	It is flexible in respect to gelation time, gel pore size, chemical functionalization, and degradation or dissolution.
Physically cross-linked hydrogels are less stable against degradation.	Chemically cross-linked hydrogels are very stable against degradation.
These are homogeneous.	These are non-homogeneous.
Physical hydrogels have hydrophilic and hydrophobic regions present in the polymeric network.	Chemical hydrogels have domains of high cross-link density as compared to conventional hydrogels.
These hydrogels show poor mechanical properties because of the reversible physical interactions.	The mechanical properties of these hydrogels are higher than physically cross-linked hydrogels.
For incorporation of bioactive substances, these gels are of great interest.	These are being used in a number of applications like pharmaceutical, agriculture, food industry, cosmetics, etc.

**Table 2:** Hydrogel types with their composition and drug incorporated.

**Types of hydrogel**	**Polymer (s)**	**Drug**	**References**
**Hydrophobic interactions**	PX 407	Benzoic acid/ p-hydroxybenzoic acid	22
	PX 188, PX 181 and Pluronic P85	2-n-undecylmalonic acid, doxorubicin, ammonium, acetic acid and tetraphenylborate.	23
	PX 407, Chitosan and Carbopol-934	Meloxicam	24
	PX 407	Indomethacin	25
	PX 407	Adriamycin and 5-flurouracil	26
	PX 407	Mitomycin C	27
	HPC and PX 407	Ketoprofen	28
	PX 407 and PX 188, Polyvinylmethylether maleic anhydride copolymer, HPMC, PE-400.	Meloxicam	30
	HPMC K100M and PX 407	Procaine	31
	HPMC and PX 407	Pranoprofen	32, 33
	HPMC and PX 407	Lidocaine HCl	34
	HPMC and PX 407	Aceclofenac and Metoprolol succinate	35, 36
	PNIPAM grafted with hyaluronic acid and chitosan	Riboflavin and 5-fluorouracil	41, 42
	Chitosan grafted with PEG 40	BSA	45
	Alginate solution	BSA	46
**Charge interactions**	HTCC and GP	Doxorubicin HCl	49
**Polyelectrolyte complexes**	Chitosan with sodium alginate, carboxymethyl cellulose sodium and k-carrageenan	Metoprolol tartrate	53
	Chitosan and gelatin	----	54
	Chitosan and poly-(γ-glutamic acid) (γ-PGA)	----	55, 56
	Alginate and chitosan	----	57
	Phosphorylated chitosan (a polycation) and chitosan	Osteoblasts	58
	Polycationic N-trimethyl chitosan and polyanionic N-carboxymethyl chitosan	Dexamethasone	59
	Quaternized chitosan and PAA	----	60
**Physical mixtures and H-bonding**	Carboxymethyl tamarind kernel polysaccharide and PVA	Metronidazole	64
	Chitosan and PVA	Minocycline and Gentamycin	66, 67
	Microcrystalline cellulose and PVA	Vanillin	68
**Stereocomplexation**	L- and D- PLA	----	70
	Isotactic and syndiotactic PMMA	----	71
	D-PLA and L-leuproide	----	72
	Dextran precursors grafted L-lactide and D-lactide oligomers	----	73
**Supramolecular chemistry**	PEO polymers and α-cyclodextrins	----	75
	β-cyclodextrin & PPO-grafted dextran	----	76
	PEO-poly(R-3-hydroxybutyrate) (PHB)-PEO triblock copolymers & α-cyclodextrin	----	77
**Cross-linking**			
**(i) Small-molecule cross-linking**	Oxidized gum arabic	Primaquine	79
	Genipin	BSA	86
	Chitosan	Oxprenolol HCl Propanolol HCl 5-Fluorouracil	87 95 89
	Chitosan–alginate	Indomethacin	90
**(ii) Polymer-polymer cross-linking**	Hyaluronic acid with amino or aldehyde functionality	Bone morphogenetic protein-2	91
	Cross-linked hyaluronic acid	Tissue plasminogen activator and budesonide	93
	Poly(aldehyde guluronate) and adipic acid dihydrazide	Bone precursor cells and growth factor	94
**(iii) Photo cross-linking**	Chitosan-pluronic	Human growth hormone (hGH) Plasmid DNA	96 97
	Modified chitosan-PEG	Growth factors and cells	98
**(iv) Enzymatic cross-linking**	Gelatin cross-linked by mTG	Regenerative cells (HEK293) Interleukin-2	100 101
	Tyramine modified hyaluronic acid by HRP	Dexamethasone	103
**Interpenetrating networks**	Chitosan, PAA and PVP	Clarithromycin	116
	Chitosan and PVP	Clarithromycin	117
	SA and PVA	Prazosin HCl	118
	Chitosan and methylcellulose	Theophylline	119
	Chitosan and PX	5-fluorouracil	120

## Conclusion and future prospective

Over the past 50 years, there has been continuous progress in designing of hydrogels that led to numerous applications such as pharmaceutical, biomedical, agrochemicals, food industry, etc. In pharmaceutical therapeutic delivery, hydrogels are available in various dosage forms such as tablets, capsules, microspheres, transdermal films, wound dressings, etc. These hydrogels are also used to develop catheter, vascular grafts, semiocclusive dressings, mammary implants, transdermal drug delivery systems, scaffold for tissue engineering and medicated patches etc.^6^ Despite the wide use, hydrogels suffer significant disadvantages such as low mechanical strength and toughness, difficulties in handling, sterilization, syringability issues. Special structural configurations like slip-link networks, nanocomposite hydrogels, double network hydrogels, multi-functional cross-linked hydrogels, and homogeneous hydrogels can be synthesized to enhance mechanical strength and toughness of resulting hydrogels.^122^ These techniques should be followed by a suitable method of polymerization or cross-linking in order to make them desirable for suitable application. Another way to enhance mechanical property along with improved biocompatibility and physical property is to combine both physical and chemical cross-linking in a single hydrogel system.

The syringability issue can be solved to a large extent by designing physical gelators which gel at lower polymer concentrations and at more precise gelation temperatures, like in-situ or thermosensitive gels, would reduce the risk of premature gelation inside the needle upon injection. Similarly, for covalently cross-linked hydrogels, the development of strategies to release cross-linker in a triggered manner inside the body, thereby minimizes the risk of syringe clogging, improve the localization of cross-linker release to minimize in vivo toxicity. This strategy is used to transform hydrogels in to “smart” materials that can respond to changes in their environment. In addition the hydrogel properties can be further modified by incorporation of micro or nano fillers. More recently, hydrogels have been modified into hydrogel microparticles for solubility enhancement purposes.^123,124^

In the present scenario, the focus needs to be shifted towards the development of innovative methods to prepare: (i) hydrophilic polymers of desirable functional groups, (ii) multifunctional/multiarm structures such as grafted or branched co-polymers and star polymers that would offer better properties and suit wider applications in the future.

## Acknowledgments

The author thanks management of GITAM University to provide necessary facilities and support.

## Ethical Issues

Not applicable.

## Conflict of Interest

The author declares no conflicts of interest.
